# Digital Health Literacy: A systematic review of interventions and their influence on healthcare access and sustainable development Goal-3 (SDG-3)

**DOI:** 10.12669/pjms.41.3.10639

**Published:** 2025-03

**Authors:** Tehreem Mukhtar, Muhammad Naveed Babur, Roohi Abbas, Asima Irshad, Qurba Kiran

**Affiliations:** 1 Tehreem Mukhtar, Ms NMPT, PhD scholar, Superior University, Lahore Pakistan; 2 Muhammad Naveed Babur, Professor/ Dean Faculty of Allied Health Science, Superior University, Lahore Pakistan; 3 Roohi Abbas, Ms NMPT, PhD scholar, Superior University, Lahore Pakistan; 4 Asima Irshad, PP-DPT, PhD scholar, Superior University, Lahore Pakistan; 5 Qurba Kiran, Ms NMPT, PhD scholar, Superior University, Lahore Pakistan

**Keywords:** Digital Health, Health Care Quality, Health Services Accessibility, Health Equity, Sustainable Development, Treatment Expectations

## Abstract

This study explores how interventions focused on digital health literacy (DHL) can improve access to healthcare and contribute to achieving Sustainable Development Goal-3 (SDG- 3). We scrutinized information from PubMed (MEDLINE), Scopus, and Web of Science released search articles from March 1, 2020 to January 31, 2024. Following the Preferred Reporting Items for Systematic Reviews and Meta-Analyses (PRISMA) guidelines, the review concentrated only on experimental studies that assessed how DHL initiatives have influenced enhancing patient health outcomes and access to healthcare. Research that did not cover DHL or the availability of healthcare, was not included. The analysis was primarily qualitative, focusing on thematic patterns and insights rather than statistical outcomes. Our results showed that DHL interventions typically result in enhanced health literacy, improved medication adherence, and higher self-confidence, particularly benefiting marginalized communities. Limitations to safe & accessible healthcare underscore the need for more focused and culturally appropriate strategies. This review shows that interventions by DHL can greatly enhance healthcare results, highlighting the need to tackle inequalities to ensure marginalized communities also benefit.

## INTRODUCTION

Health Literacy being an important component of patient-centered care refers to the patient’s education and their ability to communicate, interpret health care information, and make informed decisions, it has proven to be a drastic shift of paradigm with patients being more informed and involved in the whole process of health care provision.^1^ In the early years of the 21^st^ century, with the popularity of the internet and smartphones in businesses as well as households, Patient began to rely on information from digital media as they started having instant answers to their questions from smartphones by simply searching on Google and similar apps without confirming the authenticity of information.^2^ Therefore, the term “Digital health literacy” was coined to address all those benefits and barriers associated with technological advances that impact patient healthcare in one way or another.^3^ As defined by the World Health Organization “Digital Health Literacy signifies improvement in patient-related health outcomes with the use of Web technologies, telemedicine & tele rehab, virtual assistants for care, remote care and monitoring, use of smart devices and wearable, digital dissemination & sharing of relevant and important information.^4^

Digital Health literacy which is an extension of eHealth literacy is paving the path to attain universal health coverage by providing accessible yet sustainable health coverage, by integrating modern technological advancements within the healthcare industry.^5^ Since sustainable development goal three is a precedence towards healthy life & well-being of persons of all age groups, particularly emphasizing reduction in maternal mortality rates, cure & control of communicable diseases, and focus on mental health, it is directly linked with DHL interventions to attain the set standards of health care coverage and accessibility.^6^,^7^

In 2023 König and Suhr’s study highlights the “Apocalypse?” intervention’s effectiveness in enhancing digital health literacy and app knowledge, with positive feedback suggesting long-term benefits. Digital health literacy interventions, including web-based mobile apps and digital platforms, have been tested for their effectiveness in improving users’ ability to access reliable health information, enhancing disease outcomes.^8^

In a recent study by Pradhan et al in 2023, lifestyle modification through digital intervention in the form of culturally adapted video education training was designed for newly diagnosed diabetic patients, and results showed that video education is effective in gaining clinical outcomes.^9^ After thorough investigations and determining competencies & skills needed to explore/utilize eHealth literacy, many outcome measures were developed to address varying populations across the globe, including an outcome measure based on the eHealth literacy model Norman and Skinner, termed as “eHealth Literacy Scale” or in simple terms e-HEALS.^10^ This outcome measure was an 8-tem measure of eHealth literacy that proved to be a reliable tool to assess end-user skills to extract and use information technology, particularly young people.^11^

In this era of technological development, digital health literacy is a powerful tool to improve healthcare access and gain competitive advantages of technology in healthcare to meet universal healthcare coverage requirements. It is also a step towards attaining sustainable developmental goals of health and well-being. Therefore, the main objective of this systematic review was to examine and analyze interventions designed to enhance digital health literacy and their impact on healthcare access.

## METHODS

**Fig.1:**
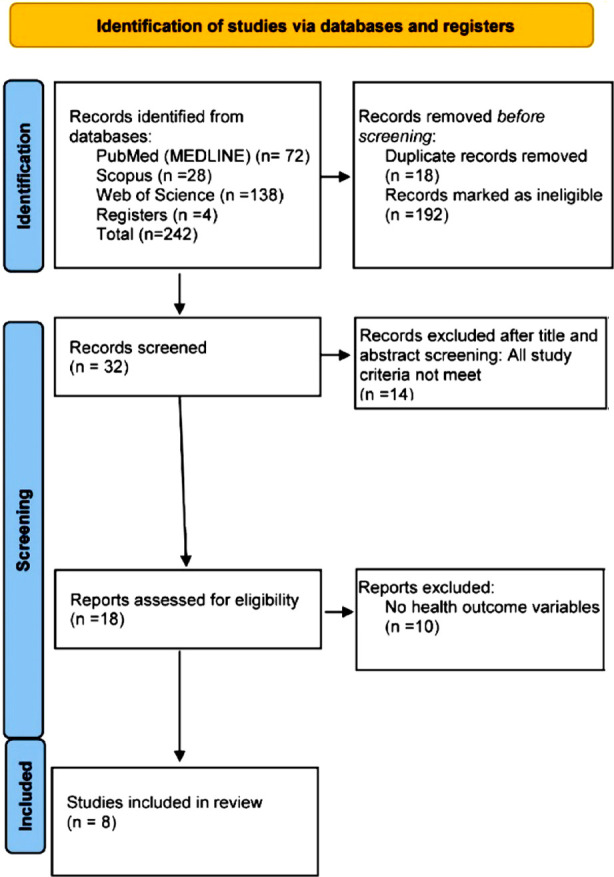
PRISMA flow diagram.

This systematic review examines the engagement of interventions and their influences on healthcare access and SDG3, as well as the effectiveness of online health in enhancing individuals’ understanding and utilization of digital health tools and resources. It was conducted as per the Preferred Reporting Items for Systematic Reviews and Meta-Analyses (PRISMA) guidelines. (Fig.1)

### Data Sources:

In February 2024, we conducted an electronic search of databases such as PubMed (MEDLINE), Scopus, and Web of Science. Articles related to the proposed research topic, which has two related concepts (concept 1: Digital Health literacy and concept 2: Influence of interventions on health care access and SDG3), were assessed for suitability for inclusion. Concept 1 terms included online courses, online training, webinars, health courses, health training, e-learning, and e-health. Concept 2 terms included health literacy, health outcomes, health measures, health status, and health knowledge. We searched the databases using free text terms or natural language terms. We developed a comprehensive search strategy using appropriate keywords and databases relevant to our topic. The Key words were: e-health literacy AND SDG3, digital health literacy AND health service, digital health service OR e-health service AND SDG3, health information service OR e-health information service. Screening the search results based on our inclusion and exclusion criteria. Initially, scan titles and abstracts to identify potentially relevant studies. We extracted relevant data from selected studies and focused on key information such as study design, population, intervention/exposure, outcomes, methodology, and results, (Table-I).

**Table I:** Summary of Studies Evaluating Digital Health Literacy Interventions and Their Impact on Health Outcomes.

Authors, Year, Reference	Title	Methodology	Result	Conclusion	Limitations	Practical Implication
Seyedeh Belin et al 2020 12	Communication skills training for physicians improve health literacy and medical outcomes among patients with hypertension: a randomized controlled trial	Randomized controlled trial on 240 hypertensive patients and 35 physicians. The intervention group received educational training, control group received routine care.	Improved physician-patient communication, hypertension outcomes, medication adherence, and self-efficacy. Better blood pressure control and enhanced health literacy skills in patients.	Physician communication training improves patient health literacy and medical outcomes. Enhanced self-efficacy and medication adherence in hypertensive patients. Training impacts counseling, health literacy, self-efficacy, and adherence positively.	Mental illness exclusion, limited age range, and informed consent issues Lack of detailed discussion on data collection guidelines and recruitment	Improved physician communication enhances patient health literacy and medical outcomes. Training physicians can lead to better hypertension control and medication adherence.
Kai Wehkamp et al 2021^13^	Enhancing Specific Health Literacy with a Digital Evidence-Based Patient Decision Aid for Hypertension: A Randomized Controlled Trial	Randomized controlled trial with 124 participants The intervention group provided with online decision aid, control group searched for information without support.	Intervention group showed a significant increase in specific health literacy for hypertension. Results were significant for understanding, appraising, and applying health-related information. Similar outcomes were observed for participants with different education levels.	Digital EbPDAs enhance specific health literacy, especially for hypertension. EbPDAs target vulnerable populations effectively, regardless of education level.	Online scenario lacks real urgency for patients’ life-changing decisions. Elevated dropout rate due to effort needed for digital EbPDA Study not powered to analyze the influence of participants’ educational level.	Digital EbPDAs enhance specific health literacy for hypertension effectively. EbPDAs target vulnerable populations and perform equally across education levels.
EunKyo Kang et al 2021^3^	Efficacy of Health Coaching and an Electronic Health Management Program: Randomized Controlled Trial	ANOVA for baseline characteristics. Generalized linear models for outcomes by group assignment.	Positive stage changes in the transtheoretical model varied among different strategies. Self-management strategy scores differed among groups, measured using SAT-SF.	Health coaching with ICT was more effective in self-management. Improvement in health habits was observed with the combined intervention.	Short intervention duration of 3 months Limited focus on a few diseases Unclear long-term sustainability of results	Health coaching with ICT improves self-management and healthy behaviors significantly. Combination of coaching and ICT enhances exercise and health habits. The intervention positively impacts chronic disease management strategies.
Szu-chi Huang et al 2022 14	Effectiveness of Tailored Rehabilitation Education in Improving the Health Literacy and Health Status of Postoperative Patients with Breast Cancer A Randomized Controlled Trial	Randomized controlled trial with 99 breast cancer patients. Tailored rehabilitation education program for health literacy and health status	TRE program improved HL and health status in breast cancer patients. No significant difference in activity scores between intervention and control group	TRE program significantly improved health literacy and health status. No significant difference in activity scores between intervention and control groups	Participants were young and highly educated, limiting generalizability. Blinding both therapists and patients was challenging to maintain.	Incorporate TRE in rehabilitation to improve health status post-surgery Establish patient-centered interventions to enhance clinical outcomes in Taiwan.
Shahina Pardha et al 2023 9	Individual patient-centered target-driven intervention to improve clinical outcomes of diabetes, health literacy, and self-care practices in Nepal: A randomized controlled trial	Random allocation into intervention and control groups for diabetes management. Culturally and linguistically appropriate diabetic video education program for training. Customized dietary and physical activity plan with specific targets. Weekly compliance monitoring via telephone calls for participants	HbA1c decreased to 6.1% in the intervention group. Intervention group had lower cholesterol and exercised more. Daily white rice consumption decreased significantly in the intervention group. All intervention group participants self-initiated retinal screening checks.	Intervention group showed significant improvements in HbA1c, cholesterol, and physical activity. Diabetic health literacy and self-care practices improved in the intervention group. Cultural and linguistically appropriate lifestyle intervention led to positive outcomes.	Lack of cultural diabetes information for specific groups in Nepal. Limited access to media for diabetic knowledge in remote areas.	Culturally tailored interventions improve diabetes outcomes and health literacy. Video education enhances diabetic self-care practices and clinical outcomes. Weekly follow-ups and reminders are crucial for sustained results. Improved health literacy leads to better uptake of healthcare services.
SR Jafree et al 2023 14	Impact of a digital health literacy intervention and risk predictors for multimorbidity among poor women of reproductive years: Results of a randomized-controlled trial	Descriptive statistics and multivariate logistic regression were used for analysis. Randomized control trial with 820 women aged 15-45 in Pakistan	The intervention group had higher confidence in health management skills. No significant improvement in hand hygiene and protective behavior outcomes. Secondary outcomes showed no significant improvement in overall health quality.	Digital health literacy aids in managing multimorbidity among women effectively. Efforts are crucial for improving maternal and child health in developing regions.	No significant improvement in hand hygiene and protective behavior outcomes. Secondary outcomes like quality of life and emotional well-being lacked improvement.	Digital health literacy aids in managing multimorbidity among women effectively. Interventions can enhance health awareness and hygiene practices in low-income settings.
Anna Quialheiro et al 2023 15	Promoting Digital Proficiency and Health Literacy in Middle-aged and Older Adults Through Mobile Devices With the Workshops for Online Technological Inclusion (OITO) Project: Experimental Study	Quasi-experimental design with nonrandomized allocation of participants in 8 workshops. Collected sociodemographic, health status, and mobile use information at baseline. Used Mobile Device Proficiency Questionnaire and Health Literacy Scale questionnaires.	Significant digital literacy improvement post-intervention, sustained after 1 month No significant change in health literacy during the project period	OITO project had high recruitment, satisfaction rates, and improved digital literacy. Participants maintained digital literacy scores post-intervention but no change in health literacy.	No significant change in health literacy during the project period. No significant difference in health literacy at T2 or T3.	Enhances digital literacy in older adults through structured workshops. Improves autonomy and digital skills with mobile device proficiency training. Sustained digital literacy gains post-intervention with Android smartphone usage.
Claudia Marisol et al 2024 16	Impact of a culturally adapted digital literacy intervention on older people and its relationship with health literacy, quality of life, and well-being	Quasi-experimental non-equivalent control group study with pre and post-evaluation. Culturally adapted multicomponent program implemented in a non-formal education context.	DL intervention showed significant improvement in personal well-being and social support. SF-12 quality of life results indicated improvement in treatment group	Culturally adapted DL intervention impacts digital literacy and well-being. No direct or indirect effects on health literacy were identified. Interventions should focus on health improvement and social integration	Study limitations include self-selection bias and potential application issues	Culturally adapted DL interventions enhance social integration and well-being. Digital literacy programs in Chile promote social inclusion and empowerment.

### Inclusion Criteria:

### Study Design:

Research papers that exclusively consist of experimental studies, whether they are randomized or non-randomized /quasi-experimental.

### Population:

Studies that focus on individuals or populations using digital health interventions.

### Intervention:

Articles assessing methods that enhance healthcare access in developing countries to aid in achieving Sustainable Development Goal-3, aimed at promoting good health and well-being. These eight research studies used various approaches to evaluate the effects of interventions on digital health literacy. Options included conventional medical evaluation techniques, self-reported patient results, Digital Evidence-Based Patient Decision Aids (EbPDAs), SF-12, and health literacy assessments. Other resources like HbA1c tests and culturally specific educational materials were utilized to assess results linked to interactions between doctors and patients, management of hypertension, self-care techniques, and understanding of healthcare information. Various techniques were used in these studies to enhance health literacy and outcomes.

The interventions offered included training for healthcare providers to improve their communication skills, evidence-based electronic tools for patient decision-making, health coaching using technology, customized education for rehabilitation, appropriate digital literacy programs for different cultures, and patient-centered interventions for managing chronic conditions like diabetes. Typically, these actions led to improved health understanding, better health management skills, and better medical care results. Nevertheless, certain studies highlighted limitations in the usefulness and lasting impact of the interventions. The study included research papers sourced from databases like PubMed (MEDLINE), Scopus, and Web of Science, containing full text published from March 1, 2020, to January 31, 2024.

### Exclusion Criteria:

### Research Studies:

Editorials, short communications, qualitative studies, case-control studies and narrative reviews without original data.

### Irrelevant Population:

Studies not focusing on digital health literacy or those that do not address healthcare access or SDG 3.

### Non-Intervention Studies:

Research that does not specifically investigate interventions designed to improve digital health literacy.

### Language and Date Restrictions:

Articles not published in English or those outside the predefined date range for the review.

### Data Analysis:

Data analysis in this systematic review was performed using a thematic synthesis approach to identify recurring patterns and insights across the included studies. This approach included the following steps:

### a) Coding of Data:

Extracted data from the studies were reviewed, and relevant information was systematically coded under predefined themes, such as intervention type, outcome measures, population characteristics, and alignment with Sustainable Development Goal-3 (SDG 3).

### b) Thematic Development:

Coded data were categorized into broader domains, including healthcare access, digital health literacy outcomes, and barriers to intervention effectiveness.

### c) Interpretation and Synthesis:

Patterns and associations between digital health literacy interventions and their impacts on healthcare access outcomes were synthesized qualitatively, with particular attention to their role in achieving SDG 3. For studies reporting quantitative outcomes, descriptive statistics (e.g., frequencies, percentages) were tabulated and summarized, while the primary analysis focused on qualitative thematic patterns rather than statistical comparisons. The review was conducted following the Preferred Reporting Items for Systematic Reviews and Meta-Analyses (PRISMA) guidelines to ensure methodological transparency and rigor.

## RESULTS

This systematic review highlights the role of digital health literacy interventions in improving healthcare access, health literacy, and clinical outcomes, aligning with Sustainable Development Goal-3 (SDG 3). Educational programs, digital tools, and community-based initiatives enhanced self-care practices, disease management, and health outcomes.

Educational programs such as e-learning and webinars improved participants’ ability to access and interpret health information. Digital tools, including mobile health applications, enhanced self-management, particularly for hypertension and diabetes, leading to improved medication adherence and blood pressure control. Community-based interventions, especially culturally adapted programs, improved digital literacy but required additional support to enhance health literacy.

Health literacy improvements were noted in participants engaging with digital literacy training, leading to better preventive care and lifestyle choices. Self-care practices improved with enhanced symptom monitoring and medication adherence. However, challenges included technological barriers, cultural adaptability, and sustainability concerns.

Digital health literacy interventions contribute to SDG 3 by improving healthcare accessibility, reducing disparities, and promoting preventive care. Continued development, culturally tailored strategies, and long-term evaluation are essential for maximizing their impact on public health and equity.

## DISCUSSION

This systematic review focused on how various interventions to enhance DHL affect healthcare access and how they support Sustainable Development Goal-3 (SDG- 3). A study of the literature combined the results of various research to understand that DHL interventions affected healthcare outcomes, access, and alignment with global health goals.

### Influence of Digital Health Intervention on health care access:

Interventions in digital health literacy have an extensive effect on access to healthcare. These approaches enable people to better understand, evaluate, and use health information by increasing knowledge related to health. People who have more knowledge are better able to understand complicated healthcare systems, make informed choices about their well-being, and interact with healthcare professionals with confidence.

A study by Tavakoly Sany SB et al. (2020) focused on educational training for physicians. This showed improved physician-patient communication, hypertension outcomes, medication adherence, and self-efficacy.^12^ These improvements indicate the value of targeted educational interventions in the hospital context and increase patient health literacy and blood pressure control. The e-learning courses emphasized by Lars König et al. (2022) in their study, showed higher health literacy levels, improved competency in theoretical and practical knowledge, critical thinking, self-awareness, and citizenship of participants.^17^ Furthermore, these therapies help people manage their health conditions on their own. Digital health literacy interventions enable people to take control over their health outcomes by educating them with the knowledge, abilities, and resources needed for active management of their health, such as tracking symptoms, keeping an eye on vital signs, and following medication regimens. Digital platforms were used in several studies to deliver health literacy treatments.

As an example, EunKyo Kang et al. (2021) improved participant health habits and self-management by combining an electronic health management tool with health coaching.^3^ Culturally adapted digital literacy intervention used by Claudia Marisol et al. (2024) impacts digital literacy and well-being but it has no direct or indirect effects on health literacy were identified.^16^ Pardha et al.’s study from 2023 showed significant improvements in HbA1c, cholesterol, and physical activity Diabetic health literacy and self-care practices improved in the intervention group.^18^ The importance of achieving sustainable development goals is great when digital health interventions and health literacy come together. These efforts may be helpful in achieving more general development goals like decreasing inequality, enhancing health and well-being, and advancing sustainable communities by connecting the digital gap, promoting health literacy.

Furthermore, by improving knowledge and promoting the use of necessary health services, digital health literacy initiatives support preventive care. Individual patient-centered target-driven intervention in Shahina Pardha et al.’s study from 2023 showed significant improvements in HbA1c, cholesterol, and physical activity Diabetic health literacy and self-care practices improved in the intervention group. Cultural and linguistically appropriate lifestyle intervention led to positive outcomes.^9^ The study’s intervention entailed Tailored Rehabilitation education to patients who had undergone surgery for breast cancer, with an emphasis on enhancing their health literacy and overall well-being during the healing process. The findings highlight the value of patient-centered therapies in helping people recover from injuries and improving their general health outcomes.^19^ These interventions showed that digital health literacy intervention has potential benefits in managing chronic diseases and it has varying impacts on different aspects of health literacy. Targeted interventions are most effective in managing specific knowledge rather than broader health literacy. These studies show that complicated health issues can be successfully addressed by combining patient-centered methods with digital technologies. We can enhance population health, reduce disparities in health care, and ultimately contribute to sustainable development by supporting digital health literacy and culturally appropriate interventions.

Kai Wehkamp et al in 2021 used online decision aid as an intervention, their study showed how useful these tools are for improving health literacy.^13^ Another study by Christine Holst and colleagues claimed free digital health education as an intervention to improve health literacy. Digital health education improves knowledge in rural low-income countries significantly.^20^ Together, these studies indicate the importance of intuitive, readily available, and culturally acceptable digital health interventions to maximize their impact on population health. It is essential to ensure equal access, address inequalities in health literacy, protect confidential information, and maintain long-term projects. Through funding research, development, and policy, we can fully utilize digital health to enhance population health.

In a study by SR Jafree et al. (2023), the impact of digital health intervention as risk predictor for multimorbidity among poor women in their reproductive years. Digital health literacy aids in managing multimorbidity among women effectively, they concluded.^14^ The intervention in a study by Anna Quialheiro et al. is the Workshops for Online Technological Inclusion (OITO) project. The OITO project had high recruitment, satisfaction rates, and improved digital literacy. Participants maintained digital literacy scores post-intervention but showed no change in health literacy.^15^ The research that are being presented show the complex relationship that exists between health literacy, digital health interventions, and achieving the Sustainable Development Goals (SDGs). The combined findings of the research by Durand et al. (2021) and Woods-Townsend et al. (2021) show that personalized interventions can successfully enhance health outcomes by filling in information gaps and empowering people, which raises the likelihood of screening, lowers inequities, and encourages healthier habits.^21^,^22^

The favorable effects on multiple medical conditions management, maternal health, and disease prevention show how much promise there is for improving health outcomes with digital platforms; yet, the studies also point out the complexities and difficulties that come with using them.

Community-focused interventions, such as those found in studies by Christian E. Vazquez et al. (2023) and Dominic Agyei Dankwah & George Clifford Yamson (2019), emphasize the value of community participation and group-based learning in advancing health literacy.^23^,^24^

A study by Sergi Blancafort Alias et al 2021 used group-based intervention delivered in low-income urban areas. Group intervention in primary care improves mental health in older adults^25^ people can be empowered to prioritize preventive measures like screenings, immunizations, and lifestyle modifications by getting tailored messaging and education. Notably, by concentrating on underprivileged and marginalized groups, these interventions also significantly contribute to resolving healthcare inequities. Digital health literacy efforts assist in closing gaps in healthcare access by customizing interventions to suit specific requirements. This guarantees that everyone has an equal opportunity to benefit from healthcare services. Additionally, by giving people the tools they need to use and understand digital health platforms and technology, digital health literacy interventions support digital inclusion.

### Role of digital health literacy interventions in contributing to the achievement of Sustainable Development Goal-3:

Ensuring healthy lives and fostering well-being for all is the third SDG and DHL interventions have altered the rules in this regard. This systematic review investigated a range of therapies, from physician training to smartphone health apps. Each of these strategies showed encouraging results in several important domains. Firstly, DHL interventions empower individuals with the knowledge and skills to manage their health. Secondly, these interventions promote preventive care by raising awareness. People become more inclined to take preventive measures like screenings and vaccinations, ultimately reducing the risk of chronic illnesses. Thirdly, tackling health equity benefits greatly from DHL interventions. Through targeted programming, these initiatives close the healthcare access gap and strengthen the power of marginalized communities.

These developments have a direct impact on accomplishing SDG 3’s main goals, which include lowering maternal mortality, preventing child deaths, fighting infectious diseases, and enhancing mental health. Achieving worldwide effect also requires creating international and culturally sensitive methods, as well as ensuring long-term access to digital tools and resources. For Pakistan, there is dire need to develop digital healthcare infrastructure, especially in Rural and underdeveloped areas to improve the access to healthcare services. By funding DHL projects in Pakistan, we can enable people and communities to take control of their health and open the door to the society that SDG 3 depicts as being healthier and more equitable. But for DHL to reach its full potential, various stakeholders must work together. Governments in Pakistan need to give DHL infrastructure investments priority, create laws that support it, and guarantee that everyone has access to resources for digital health. Healthcare providers should work with patients to collaborate on digital health options, train staff in digital literacy, and incorporate DHL into their offerings. Scholars must persist in studying the effectiveness of various DHL treatments, identify obstacles to implementation, and formulate culturally suitable approaches. Together, researchers, policymakers, and healthcare professionals can build a future where DHL enables people to take control of their health and lead better, more equal lives, as planned by SDG 3.

### Limitations:

The review’s findings are constrained by the short duration of many interventions, which limits insights into their long-term impact. Variability in study designs and outcome measures also affects the comparability and generalizability of the results. Additionally, disparities in digital access and cultural contexts may influence the effectiveness and applicability of digital health literacy interventions.

## CONCLUSION

DHL interventions are effective in enhancing healthcare access and aligning with Sustainable Development Goal-3, improving health management and outcomes. However, addressing limitations such as intervention duration, methodological variability, and digital access disparities is essential for optimizing their impact and ensuring broad applicability.

### Author’s Contribution:

**TM** and **RA:** Came up with the idea, planned, and performed the data extraction, compiling, composing, and revising of manuscript.

**AI** and **QK:** Were responsible for collecting the data and writing the manuscript, creation, writing, and revision of the manuscript.

**NB:** Is the guarantor and is accountable for all aspects of the work in ensuring that questions related to the accuracy or integrity of any part of the work are appropriately reported.
